# High-Dimensional Descriptor Selection and Computational QSAR Modeling for Antitumor Activity of ARC-111 Analogues Based on Support Vector Regression (SVR)

**DOI:** 10.3390/ijms13011161

**Published:** 2012-01-20

**Authors:** Wei Zhou, Zhijun Dai, Yuan Chen, Haiyan Wang, Zheming Yuan

**Affiliations:** 1 Hunan Provincial Key Laboratory of Crop Germplasm Innovation and Utilization, Changsha 410128, China; E-Mails: mengrzhou@163.com (W.Z.); daizhijun@foxmail.com (Z.D.); chenyuan0510@126.com (Y.C.); 2 Hunan Provincial Key Laboratory for Biology and Control of Plant Diseases and Insect Pests, College of Bio-Safety Science & Technology, Hunan Agricultural University, Changsha 410128, China; 3 Department of Statistics, Kansas State University, Manhattan, KS 66506, USA; E-Mail: hwang@ksu.edu

**Keywords:** ARC-111 analogues, QSAR, support vector regression, high-dimensional descriptor selection nonlinearly (HDSN) method, worst descriptor elimination multi-roundly (WDEM) method, RPMI8402

## Abstract

To design ARC-111 analogues with improved efficiency, we constructed the QSAR of 22 ARC-111 analogues with RPMI8402 tumor cells. First, the optimized support vector regression (SVR) model based on the literature descriptors and the worst descriptor elimination multi-roundly (WDEM) method had similar generalization as the artificial neural network (ANN) model for the test set. Secondly, seven and 11 more effective descriptors out of 2,923 features were selected by the high-dimensional descriptor selection nonlinearly (HDSN) and WDEM method, and the SVR models (SVR3 and SVR4) with these selected descriptors resulted in better evaluation measures and a more precise predictive power for the test set. The interpretability system of better SVR models was further established. Our analysis offers some useful parameters for designing ARC-111 analogues with enhanced antitumor activity.

## 1. Introduction

Topoisomerase I (TOP I) is a clinical target for the treatment of cancer [1]. Camptothecin (CPT) and several CPT derivatives (e.g., CPT-11, topotecan) have been developed for clinical use due to CPT-induced TOP I inhibition, referred to as a cleavage complex. Despite their potential, CPTs are chemically unstable, and are substrates for the ATP-binding cassette (ABC) transporter breast cancer resistance protein (BCRP) known to be expressed in many human tumors, which bind to human serum albumin (HAS) in their carboxylate forms, leading to reduced potency in humans compared to mice [2]. So it is necessary and important to develop alternative TOP I targeting agents. 8,9-Dimethoxy-5-(2-*N*,*N*-dimethylaminoethyl)-2,3-methylenedioxy-5*H*-dibenzo[*c*,*h*][1,6] naphthyridin-6-one (ARC-111) is a promising new TOP I-targeting antitumor drug with a different drug resistance profile [2]. Cytotoxicity of ARC-111 in RPMI 8402 tumor cells has been proved to be correlated with TOP I-targeting activity, so ARC-111 is thought to be one of the assessment indicators for antitumor activities [3].

The quantitative structure-activity relationship (QSAR) is a powerful approach used for studying the relationship between drug activities and molecular structures, and it is helpful to explain how structural features determine drug activities. Especially, an acceptable QSAR has the advantages of higher-speed and lower-costs than experimental testing for drug activity evaluation. Yu *et al*. have compared QSAR modeling of antitumor activity of ARC-111 analogues using stepwise multiple linear regression (stepwise MLR), partial least squares (PLS) and artificial neural network (ANN), and the results showed the ANN model was the most powerful for the test [4]. However, the ANN model still had an obvious defect in the reliability of structural information because its independent variables had to be selected by linear techniques from only 15 molecular descriptors, so the QSAR of an increasing number of ARC-111 analogues possessing antitumor activities are still not well understood. Therefore, selecting more effective molecular features from the high-dimensional ones of ARC-111 analogues using new methods will possibly provide more useful information for the design of new antitumor drugs. Parameter Client provides an interface for different programs that calculate several groups of descriptors with a total number of >3000 [5]. For each ARC-111 analogue, its high-dimensional descriptors could be calculated freely and quickly. Because many of these descriptors are redundant and sometimes irrelevant, models for nonlinear selection of the most useful subset of descriptors are needed for theoretical analysis and for practical applications.

The support vector machine (SVM) is a class of learning-based nonlinear modeling technique with proven performance in a wide range of practical applications [6]. Originally, SVMs were developed for classification or qualitative modeling problems. With the introduction of a *ɛ*-insensitive loss function, SVM has been extended to solve nonlinear regression (or quantitative modeling) problems. To select reasonable features, we employed two in-house developed methods based on SVM regression (SVR): the worst descriptor elimination multi-roundly (WDEM) [7] and the high-dimensional descriptors selection nonlinearly (HDSN) [8], and then constructed QSAR models of ARC-111 analogues based on the SVR technique in this study.

The objectives of this work were: (1) to test the effectiveness of the SVR model on ARC-111 analogues by comparing them with other chemometric tools including stepwise MLR, PLS, and ANN; (2) to construct and evaluate QSAR models using SVR with selection of descriptors from high-dimensional features of ARC-111 analogues; (3) to analyze the explanatory power of the SVR models; and (4) to predict the activities of several theoretical drugs based on our model and thus provide specific parameters for future drug development.

## 2. Results and Discussion

### 2.1. Comparative QSAR Modeling with the Low-Dimensional Literature Descriptors Using Stepwise MLR, PLS, ANN and SVR Techniques

To verify the generalization ability of QSAR constructed using SVR technique, a low-dimensional literature dataset with 9 descriptors was adopted. The 9 descriptors were the combined set of features from stepwise MLR and PLS in [4]. To further eliminate the redundant descriptors from this literature dataset, every available descriptor were gradually removed one by one from the model using our WDEM method (10-fold cross-validation) until the model with the lowest *MSE* was obtained. Six key descriptors [*MW*, *Dipole*, *MolPol*, *JGT*, *E(H-bond)* and *Δ H_f_*^0^] were reserved by 3 rounds of nonlinear selection. Then the two low-dimensional datasets with 9 and 6 descriptors, respectively, were trained by leave-one-out (LOO) cross-validation and modeled in five Kernel functions (*t* = 0; *t* = 1, *d* = 2; *t* = 1, *d* = 3; *t* = 2; *t* = 3). The results of the independent test showed (1) the SVR1 model (*t* = 1, *d* = 3) with all literature features had higher predictive ability than stepwise MLR and PLS; and (2) the SVR2 model (*t* = 2) with *MSE* of 0.061, *R*^2^ of 0.950 and *R* *_pred_*^2^ of 0.918 for the test set had comparable predictive ability with the ANN (the number of units in hidden layers was four and the number in the training set was ten [4]) model even though SVR2 used less descriptors (Table 1). It indicated the SVR model was also a powerful technique for a given set of low-dimensional descriptors.

The SVR model with 6 descriptors (SVR2) produced better results than the SVR model with all 9 descriptors (SVR1). We noted that the 6 descriptors were obtained with the WDEM from the 9 descriptors. This showed that the WDEM method might be effective to choose relevant descriptors for more accurate prediction of the activities of ARC-111 analogues. This property will be helpful for the modeling with high-dimensional features. Considering nonlinear function, predictive ability and computing time, the Radial Basis Function (*t* = 2) and 10-fold cross-validation will be adopted in future feature selecting, and the Radial Basis Function (*t* = 2) and LOO cross-validation will be adopted in independent tests.

### 2.2. QSAR Modeling with the High-Dimensional Descriptors Using SVR Technique

To improve drug design of ARC-111 analogues, the analysis of high-dimensional descriptors may result in better prediction. Using the software, PCLIENT, 2,923 molecular descriptors were calculated. Then the high-dimensional dataset containing the independent variables (all 2,923 descriptors) and the dependent variables [pIC_50 (expt.)_ values] was used for modeling. Because the high-dimensional descriptors had more redundant information, we focused on how to select nonlinearly less but more critical descriptors using SVR. We have developed two novel methods that could select important descriptors from thousands of them. By initial coarse screening using the HDSN method to filter out irrelevant features, the data set would switch from high-dimensional into low-dimensional. Then further careful screening using the WDEM method would turn the data set with low-dimensional features into one with only important descriptors. Throughout the process, the descriptors in modeling with higher *MSE* values were removed gradually and nonlinearly until the model with the lowest *MSE* value was obtained. Finally, the SVR models for the test set based on the obtained descriptors were developed and evaluated.

In feature screening, the Radial Basis Function (*t* = 2) and 10-fold cross-validation were adopted. Based on our HDSN method, descriptors of 18 ARC-111 analogues in SVR3 (and SVR4) model were reduced from 2,923 to 9 (and 13) by 9 (and 8) rounds of nonlinear screening. Furthermore, based on our WDEM method, descriptors were further reduced to 7 (and 11) by 2 rounds of nonlinear screening (Table 2). In the independent test, five Kernel functions and LOO cross-validation were adopted. Finally, the effective SVR3 and SVR4 models were obtained only by the Radial Basis Function (*t* = 2). The results of the independent test (Table 2) showed the SVR3 (and SVR4) models had similar or better predictive power with *MSE* of 0.032 (and 0.028), *R*^2^ of 0.964 (and 0.971) and *R_pred_*^2^ of 0.957 (and 0.962) for the test set than stepwise MLR, PLS and ANN techniques. By nonlinear screening using our HDSN and WDEM methods, the SVR model with the obtained features from high-dimensional features of ARC-111 analogues had stronger generalization ability than all reference models for antitumor activity prediction in RPMI 8402. Furthermore, based on the SVR4 model, pIC_50 (pred.)_ values of 12 theoretical ARC-111 analogues were predicted for drug activity evaluation. The results showed no drug with higher antitumor activity appeared in these theoretical designs, and suggested utilizing other substituents or other positions to design more effective drugs.

The SVR3 and SVR4 models predicted that the antitumor activity of ARC-111 analogues depends on 7 and 11 molecular factors, respectively. According to the interpretability analysis of the SVR model we have established [9], the significance of the regression model and the importance of single indicator was obtained based on SVR and *F*-test. The results showed the nonlinear regression of the SVR3 model (*R*^2^ = 0.947) was highly significant because its *F* value (21.017) was greater than *F*_0.01_(7, 10) value, and the nonlinear regression of the SVR4 model (*R*^2^ = 0.947) was significant because its *F* value (7.310) was greater than *F*_0.05_(11, 6) value. The five most important descriptors in SVR3 were *c6A* (highly significant), *ATS1v* (highly significant), *nCIC* (highly significant), *MATS3e* (highly significant) and *nCrs* (significant), and the only one most important descriptor in SVR4 was *BELv2* (significant) (Table 3).

The *F*-test values of the independent variables showed that GSFRAG, 2D autocorrelations and constitutional descriptors, played important roles in describing anticancer activities. According to the analysis of single indicator importance, *c6A*, *ATS1v*, *nCIC*, *MATS3e* and *nCrs* in the SVR3 model and *BELv2* in the SVR4 model appeared to be the most significant descriptors of ARC-111 analogues. *ATS1v* [10], *nCIC* [11], *MATS3e* [12–16], *nCrs* [17–23] and *BELv2* [10,24–26] have been previously reported in different literature models, respectively. To our knowledge, *c6A* has never been reported as a critical descriptor, so it is unclear what new information is added as an important descriptor. Previous works have shown the physical and biological significance of several significant descriptors founded in our analysis. *nCIC*, as one of the highly significant descriptors, appears to have an influence on binding. It is likely that the active site of a possible target possesses more than one binding site, therefore the number of rings could be important for fitting into a hydrophobic pocket [11]. *MATS3e*, as one of the highly significant descriptors, are weighted by atomic Sanderson electronegativities, and might partly influence the drug aqueous solubility [15]. *BELv2*, weighted by atomic van der Waals volumes of Burden matrix, contribute to decrease the affinity of the ligands [25].

**Figure 1:**
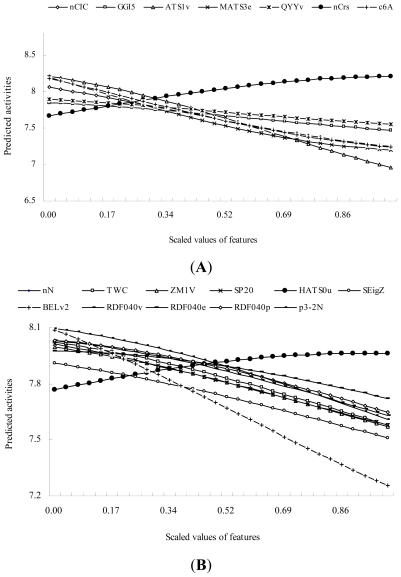
Single-factor effects of features in the SVR3 (**A**) and SVR4 (**B**) models.

For all descriptors, the analysis of single-factor effects showed that the antitumor activity was positively correlated with *nCrs* values but negatively correlated with a further 6 descriptor values in the SVR3 model, and antitumor activity was positively correlated with *HATS0u* values but was negatively correlated with the values of a further 10 descriptors in the SVR4 model (Figure 1).

Perhaps, starting from a descriptor pool and then revealing the physicochemical properties of a limited number of selected descriptors, as seen in some papers, can lead to a compromise between both approaches. In most of the models for prediction, theoretical molecular descriptors were used. Experimental chromatographic descriptors could be useful but are tedious to determine and therefore less popular [10]. Therefore, our results can be helpful to explain how descriptors could determine the antitumor activities of ARC-111 analogues, and improve drug design for new drug development. In addition to anticancer bioactivity [27], the structure activity relationship analysis can be applied to toxicology [28–30], *etc*. Therefore, a good QSAR model has broad application prospects.

## 3. Materials and Methods

### 3.1. Structures and Activities

**Figure 2:**
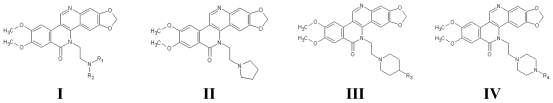
Four types of ARC-111 analogues structures.

According to the types and roles of ARC-111 substituents reported in literature [3], 12 theoretical ARC-111 analogues were designed and evaluated. The structures of these 12 theoretical analogues and 22 experimental ones from [4] were divided into four types (Figure 2) and listed in Table 4. IC_50_ (μM), the concentration of compounds causing 50% cell growth inhibition against tumor cell lines [3], are converted to negative logarithms of IC_50_ (pIC_50_) [4]. The collected 22 experimental pIC_50_ [pIC_50 (expt.)_] values against RPMI8402 tumor cells ranged from 6.071 to 9.523. To obtain statistically robust QSAR models and compare with the results of MLR, PLS, and ANN in [4], the experimental data sets in Table 4 were partitioned into the training set with 18 compounds and the test set with 4 compounds as in [4].

### 3.2. Calculation of Molecular Descriptors

First, to understand the QSAR reliability of modeling ARC-111 analogue activities using SVR technique, 4 electronic [*Dipole*, *E*(*H-bond*), *Δ H_f_*^0^ and *E*_T_], 2 spatial (*MW*, *R_g_*) and 1 physicochemical (*MolPol*) descriptors as well as different topological parameters (*JGT*, *Wiener*) from the literature were adopted to construct QSAR models. The 9 descriptors were obtained by molecule energy optimization using MM2 ChemOffice 2005, and then were calculated by MODEL and ChemOffice 2005 [4].

Second, to develop a better QSAR model based on high-dimensional data sample using SVR technique, molecular structures were represented by about 3,000 molecular descriptors that encoded much more structural information. These descriptors were generated by the software PCLIENT (http://www.vcclab.org/lab/pclient/) and classified under 24 groups (Table 5) [5]. The calculation process of the descriptors involved the following steps: the structures of the compounds were drawn using JME Editor of Peter Ertl and saved as SMILES files, and then the SMILES files as a task were added to the software PCLIENT for calculating all of the descriptors in the default state.

### 3.3. Model Development

To reduce dimensionality and improve model robustness in QSAR analysis, high-dimensional features would be screened coarsely and nonlinearly into low-dimensional features with lower mean squared error (*MSE*) by our HDSN method [8], and then low-dimensional features would be further screened nonlinearly by our WDEM method [31].

### 3.4. Model Evaluation

The selection of descriptors and the optimization of Kernel functions parameters were examined by 10-fold or LOO validation with the minimum *MSE*; the predictive capacity of the models was assessed based on *MSE*, the squared multiple correlation coefficient (*R*^2^) and the squared predictive correlation coefficient (*R_pred_*^2^) values calculated by the following equations:

(1) $$MSE=\frac{\sum {\left(y_{i}-{\hat{y}}_{i}\right)}^{2}}{n}$$

(2) $$R^{2}=\frac{\sum {\left(y_{i}-\bar{y}\right)}^{2}{\left({\hat{y}}_{i}-\bar{\hat{y}}\right)}^{2}}{\sum {\left(y_{i}-\bar{y}\right)}^{2}\cdot \sum {\left({\hat{y}}_{i}-\bar{\hat{y}}\right)}^{2}}$$

(3) $$R_{pred}^{2}=1-\frac{\sum {\left(y_{i}-{\hat{y}}_{i}\right)}^{2}}{\sum {\left(y_{i}-{\bar{y}}_{training}\right)}^{2}}$$

Here *y_i_*, *ŷ_i_*, *ȳ*, *ŷ̄* and *n*, respectively, represented the experimental values, the predicted values, the mean values of the experimental values, the mean values of the predicted values and the number of compounds of the test set, and *ȳ_training_* was the mean activity value of the training set. Generally, an acceptable QSAR model was considered to have a higher predictive power only having the lower *MSE* [31], the higher *R*^2^ [8] and the higher *R_pred_*^2^ (at least >0.6) [32] for the test set.

## 4. Conclusions

In our QSAR analysis, the structural information of 34 ARC-111 analogues was described using 2923 molecular descriptors obtained. Two groups of more important descriptors were obtained using two nonlinear descriptor selection methods, and then used to model the activities of these ARC-111 analogues based on SVR. The two SVR models demonstrated consistently better performance than reference models in terms of prediction accuracy for the test data. Our results offer new theoretical tools for drug design and development.

## Figures and Tables

**Table 1:** Comparative quantitative structure-activity relationship (QSAR) modeling of the independent test, based on the literature dataset.

	Stepwise MLR	PLS	ANN	SVR1	SVR2
Number of descriptors	5	7	9	9	6
*MSE*	0.201	0.167	0.050	0.141	0.061
*R*^2^	0.910	0.890	0.962	0.937	0.950
*R*^2^	0.730	0.775	0.933	0.811	0.918

**Table 2:** Comparative QSAR modeling of the independent test based on the high-dimensional descriptors selection using support vector regression (SVR).

	Stepwise MLR	PLS	ANN	SVR3	SVR4
Number of descriptors	5	7	9	7	11
*MSE*	0.201	0.167	0.050	0.032	0.028
*R*^2^	0.910	0.890	0.962	0.964	0.971
*R_pred_*^2^	0.730	0.775	0.933	0.957	0.962

**Table 3:** The retained descriptors by the high-dimensional descriptor selection nonlinearly (HDSN) and worst descriptor elimination multi-roundly (WDEM) methods and their *F*-test values.

Model	Group name	Descriptor name	*F*-value
SVR3	GSFRAG	*c6A*: Number of fragments Cyc6[A]	26.555 **
	2D autocorrelations	*ATS1v*: Broto-Moreau autocorrelation of a topological structure - lag 1 / weighted by atomic van der Waals volumes	25.175 **
	Constitutional descriptors	*nCIC*: Number of rings	12.210 **
	2D autocorrelations	*MATS3e*: Moran autocorrelation - lag 3 / weighted by atomic Sanderson electronegativities	12.114 **
	Functional group counts	*nCrs*: Number of ring secondary C(sp3)	5.898 *
	Topological charge indices	*GGI5*: Topological charge index of order 5	3.687
	Geometrical descriptors	*QYYv*: Qyy COMMA2 value / weighted by atomic van der Waals volumes	2.387
SVR4	BCUT descriptors	*BELv2*: Lowest eigenvalue n. 2 of Burden matrix / weighted by atomic van der Waals volumes	11.382 *
	GSFRAG-L	*p3-2N*: Number of fragments Path3 with label N on atom 2	3.771
	Randic molecular profiles	*SP20*: Shape profile no. 20	3.511
	Eigenvalue-based indices	*SEigZ*: Eigenvalue sum from Z weighted distance matrix (Barysz matrix)	2.456
	Constitutional descriptors	*nN*: Number of Nitrogen atoms	2.456
	RDF descriptors	*RDF040v*: Radial distribution function - 4.0 / weighted by atomic van der Waals volumes	2.435
	Walk and path counts	*TWC*: Total walk count	2.425
	RDF descriptors	*RDF040p*: Radial distribution function - 4.0 / weighted by atomic polarizabilities	2.398
	Topological descriptors	*ZM1V*: first Zagreb index by valence vertex degrees	2.084
	RDF descriptors	*RDF040e*: Radial distribution function - 4.0 / weighted by atomic Sanderson electronegativities	1.304
	GETAWAY descriptors	*HATS0u*: Leverage-weighted autocorrelation of lag 0 / unweighted	0.599

* *p* < 0.05;

** *p* < 0.01; *F*_0.05_(1,10) = 4.96; *F*_0.01_(1,10) = 10.04; *F*_0.05_(1,6) = 5.99; *F*_0.01_(1,6) = 13.74; *F*_0.05_(7,10) = 3.14; *F*_0.01_(7,10) = 5.2; *F*_0.05_(11,6) = 4.03; *F*_0.01_(11,6) = 7.8.

**Table 4:** Substituents and activities of 34 ARC-111 analogues.

Experimental drugs							Theoretical drugs						
	
Compound	Type	Substituent				pIC_50 (expt.)_	Compound	Type	Substituent				pIC_50 (pred.)_^b^
	
		R_1_	R_2_	R_3_	R_4_				R_1_	R_2_	R_3_	R_4_	
1	I	Me	Me			8.699	1	I	Me	Et			8.651
2		Me	Bn			7.276	2		Me	*t*-Bu			8.172
3		Et	Bn			7.114	3		Et	*t*-Bu			7.876
4		*i*-Pr	Bn			6.523	4		*t*-Bu	*t*-Bu			7.388
5		*t*-Bu	Bn			6.071	5		*t*-Bu	*i*-Pr			6.908
6		Bn	Bn			6.420	6	III			Bn		6.668
7		Et	Et			8.222	7				Et		7.208
8		*i*-Pr	*i*-Pr			8.097^a^	8				*t*-Bu		6.904
9		H	Me			9.523	9				*i*-Pr		6.617
10		H	Et			8.699^a^	10	IV				Et	6.248
11		H	*i*-Pr			8.523	11					*t*-Bu	6.102
12		H	*t*-Bu			8.699	12					*i*-Pr	6.100
13		H	Bn			7.796							
14		H	H			8.398							
15		Me	*i*-Pr			8.097^a^							
16		Et	*i*-Pr			8.301							
17	II					8.523							
18	III			H		8.155							
19				Me		7.523							
20	IV				Bn	6.398^a^							
21					H	7.046							
22					Me	6.523							

a Four experimental compounds in the test set;

b predicted values of 12 theoretical compounds by the SVR4 model.

**Table 5:** Group and count of descriptors from the software PCLIENT.

Group No.	Group of descriptors	Count	Group No.	Group of descriptors	Count
1	Constitutional descriptors	48	13	RDF descriptors	150
2	Topological descriptors	119	14	3D-MoRSE descriptors	160
3	Walk and path counts	47	15	WHIM descriptors	99
4	Connectivity indices	33	16	GETAWAY descriptors	197
5	Information indices	47	17	Functional group counts	121
6	2D autocorrelations	96	18	Atom-centered fragments	120
7	Edge adjacency indices	107	19	Charge descriptors	14
8	BCUT descriptors	64	20	Molecular properties	28
9	Topological charge indices	21	21	ET-state Indices	>300
10	Eigenvalue-based indices	44	22	ET-state Properties *	3
11	Randic molecular profiles	41	23	GSFRAG Descriptor	307
12	Geometrical descriptors	74	24	GSFRAG-L Descriptor	886
Total:					>3000

* This group of descriptors did not exist in the default state.
